# Meta‐analysis of fenestrated endovascular aneurysm repair *versus* open surgical repair of juxtarenal abdominal aortic aneurysms over the last 10 years

**DOI:** 10.1002/bjs5.50178

**Published:** 2019-05-17

**Authors:** A. D. Jones, M. A. Waduud, P. Walker, D. Stocken, M. A. Bailey, D. J. A. Scott

**Affiliations:** ^1^ The Leeds Vascular Institute Leeds General Infirmary Leeds UK; ^2^ The Leeds Institute of Cardiovascular and Metabolic Medicine, School of Medicine University of Leeds Leeds UK; ^3^ The Leeds Institute of Clinical Trials Research University of Leeds Leeds UK

## Abstract

**Background:**

Juxtarenal abdominal aortic aneurysms pose a significant challenge whether managed endovascularly or by open surgery. Fenestrated endovascular aneurysm repair (FEVAR) is now well established, but few studies have compared it with open surgical repair (OSR). The aim of this systematic review was to compare short‐ and long‐term outcomes of FEVAR and OSR for the management of juxtarenal aortic aneurysms.

**Methods:**

A literature search was conducted of the Ovid Medline, EMBASE and PubMed databases. Reasons for exclusion were series with fewer than 20 patients, studies published before 2007 and those concerning ruptured aneurysms. Owing to variance in definitions, the terms ‘juxta/para/suprarenal’ were used; thoracoabdominal aortic aneurysms were excluded. Primary outcomes were 30‐day/in‐hospital mortality and renal insufficiency. Secondary outcomes included major complication rates, rate of reintervention and rates of endoleak.

**Results:**

Twenty‐seven studies were identified, involving 2974 patients. Study designs included 11 case series, 14 series within retrospective cohort studies, one case–control study and a single prospective non‐randomized trial. The pooled early postoperative mortality rate following FEVAR was 3·3 (95 per cent c.i. 2·0 to 5·0) per cent, compared with 4·2 (2·9 to 5·7) per cent after OSR. After FEVAR, the rate of postoperative renal insufficiency was 16·2 (10·4 to 23·0) per cent, compared with 23·8 (15·2 to 33·6) per cent after OSR. The major early complication rate following FEVAR was 23·1 (16·8 to 30·1) per cent *versus* 43·5 (34·4 to 52·8) per cent after OSR. The rate of late reintervention after FEVAR was higher than that after OSR: 11·1 (6·7 to 16·4) *versus* 2·0 (0·6 to 4·3) per cent respectively.

**Conclusion:**

No significant difference was noted in 30‐day mortality; however, FEVAR was associated with significantly lower morbidity than OSR. Long‐term durability is a concern, with far higher reintervention rates after FEVAR.

## Abstract (es)

**Antecedentes:**

Los aneurismas de la aorta abdominal yuxtarrenal plantean un gran reto sobre si tratarlos de forma endovascular o mediante cirugía abierta. La reparación del aneurisma con endoprótesis fenestrada (*fenestrated endovascular aneurysm repair*, FEVAR) no esta consolidada, sin embargo, algunos pocos estudios, la comparan con la reparación quirúrgica por vía abierta (*open surgical repair*, OSR). El objetivo de esta revisión sistemática fue comparar los resultados a corto y largo plazo de FEVAR y OSR para el tratamiento de los aneurismas aórticos yuxtarrenales.

**Métodos:**

Se llevó a cabo una búsqueda de la literatura en las bases de datos Ovid Medline, EMBASE y Pubmed. Las razones para exclusión fueron series con menos de 20 pacientes, aquellas publicadas antes de 2007 y los trabajos sobre aneurismas rotos. Debido a las diferencias en las definiciones, se utilizaron los términos “yuxta/para/suprarrenal”; se excluyeron los aneurismas de la aorta tóracoabdominal. Los resultados primarios fueron la mortalidad a 30 días/intrahospitalaria y la insuficiencia renal. Los resultados secundarios incluyeron las tasas de complicaciones mayores, tasa de reintervención y tasas de fugas internas.

**Resultados:**

Se identificaron un total de 27 estudios, que incluían 2.974 pacientes. Los diseños de los estudios incluían 11 series de casos, 12 estudios de cohortes retrospectivos, un estudio caso‐control y un único ensayo no aleatorizado prospectivo. La mortalidad postoperatoria precoz agrupada tras FEVAR fue del 3,3% (i.c. del 95% 2,0‐5,0), comparado con el 4,2% (i.c. del 95% 2,9‐5,7) tras OSR. Después de FEVAR, la tasa de insuficiencia renal postoperatoria fue del 16,2% (i.c. del 95% 10,4‐23,0) comparada con el 23,8% (i.c. del 95% 15,2‐33,6) después de OSR. La tasa de complicaciones mayores precoces tras FEVAR fue del 23,1% (i.c. del 95% 16,8‐30,1) comparada con el 43,5% (i.c. del 95% 34,4‐52,8) después de OSR. La tasa de reintervención tardía tras FEVAR fue superior que tras OSR: 11,1% (i.c. del 95% 6,7‐16,4) y 2,0% (i.c. del 95% 0,6‐4,3), respectivamente.

**Conclusión:**

No se observaron diferencias significativas en la mortalidad a los 30 días, sin embargo, FEVAR presentó una morbilidad significativamente menor que OSR. La durabilidad a largo plazo es una preocupación con muchas mayores tasas de reintervención después de FEVAR.

## Introduction

Abdominal aortic aneurysms (AAAs) involving, or in close proximity to, the renal arteries pose a significant challenge during both open surgical repair (OSR) and endovascular aneurysm repair (EVAR)^1^. In 2010, the Society for Vascular Surgery *Ad Hoc* Committee on TEVAR (thoracic EVAR) released the classification of AAA and thoracoabdominal aortic aneurysm (TAAA)^2^. It defined juxtarenal aneurysms as those with no normal aorta between the upper

extent of the aneurysm and the renal arteries. The term suprarenal AAA describes aneurysms that extend above the renal arteries, but do not involve the thoracic aorta^3^. Crawford and colleagues^1^ highlighted the additional challenges faced during OSR when the aneurysm extends to the renal arteries. These include additional exposure (ligation of the left renal vein, dissection of the renal arteries and superior mesenteric artery (SMA), or a retroperitoneal approach and mobilization of the left kidney), proximal aortic cross‐clamp placement (between the renal arteries and the SMA or above the coeliac trunk), and renal artery reconstruction or reimplantation. Rates of early mortality and haemodialysis in this series were both 8 per cent. Prolonged clamp time appeared to be an independent predictor of renal failure^1^. Modern outcomes are not dissimilar to those described by Crawford *et al.* ^1^. Deery and co‐workers^4^ found 30‐day mortality rates to be three times higher after repair of juxtarenal AAA compared with standard infrarenal AAA intervention.

The morphology of a juxtarenal AAA makes it unsuitable for conventional EVAR (outside instructions for use) owing to the lack of a suitable proximal landing zone^5^. This has driven modifications to the EVAR technique in recent years, including development of the chimney EVAR (ChEVAR) (also known as the snorkel EVAR) and fenestrated EVAR (FEVAR), to provide an endovascular solution for these complex aneurysms. The literature supports the use of ChEVAR for urgent or bailout procedures but not for elective work^6^, ^7^, supported by a recent meta‐analysis^8^ that showed FEVAR to have lower 30‐day mortality, long‐term mortality and adverse renal events than ChEVAR. The aim of FEVAR is to exclude the aneurysm from the circulation while maintaining normal perfusion of the renal arteries, SMA and the coeliac trunk. FEVAR requires custom‐made stent grafts, with fenestrations created in the stent graft to match the ostia of the visceral vessels. The graft is inserted and oriented so that the fenestrations correspond to the target vessels. The visceral vessels are cannulated, and covered stent grafts are placed to provide a conduit between the main graft and the visceral arteries. Randomized trials^9^, ^10^ have compared EVAR and OSR in the treatment of infrarenal AAA and found EVAR to be associated with lower early morbidity and mortality. No such trial data exist with regard to the open or endovascular treatment of juxtarenal and suprarenal AAA. The aim of this systematic review was to examine the literature from the last 10 years for both FEVAR and OSR in the treatment of juxtarenal and suprarenal AAA.

## Methods

A literature search was conducted of the Ovid Medline and EMBASE databases to April 2017. Search terms included ‘fenestrated endovascular repair’, ‘aneurysm’, ‘open repair’ and ‘juxta‐renal aneurysm’. A second search of PubMed was conducted, using the same search terms.

### Inclusion and exclusion criteria

Prospective and retrospective cohort studies, as well as case series involving more than 20 patients, published from 2007 onwards were included. Registry data were excluded to avoid duplication of data. When duplicate data were present, the most recent, complete findings were included for analysis. Studies pertaining to all forms of complex AAA were selected, including juxtarenal, pararenal and suprarenal. Owing to variance in definitions, the terms ‘juxta/para/suprarenal’ were considered sufficient for inclusion; strict anatomical definitions were not mandated. TAAA were excluded, including Crawford type IV. Where studies presented data from TAAA that could not be analysed separately from complex AAA, they were excluded. Only elective repairs were included, thereby excluding ruptures. When considering FEVAR, only series describing custom‐made fenestrated devices were included. This excluded ‘off the shelf’ devices and physician‐modified grafts. All other forms of endovascular repair, including chimney and snorkel repairs and branched devices, were excluded. All forms of OSR were included, including transperitoneal and retroperitoneal approaches, and all variations of visceral vessel reconstruction.

### Outcomes

The primary outcomes were 30‐day/in‐hospital mortality and postoperative renal insufficiency. Secondary outcomes included major complication rates, postoperative permanent dialysis, rate of reintervention, long‐term survival and rates of endoleak. Definitions of renal insufficiency, acute kidney injury (AKI) and postoperative complications were as per individual study definition. This resulted in a heterogeneous definition of renal insufficiency, ranging from a serum creatinine rise of 30 per cent to a reduction in estimated glomerular filtration rate (eGFR) of more than 50 per cent. Major postoperative complications were defined as per individual

study definitions, and included cardiac, pulmonary, renal, mesenteric, neurological and surgical‐site complications, and return to theatre. Secondary reinterventions were defined as any intervention, whether open or endovascular, performed during the period of follow‐up for aneurysm or graft‐related complications.

### Statistical analysis

The rates of each outcome were calculated per study using a meta‐analysis of proportions using MedCalc® statistical software (MedCalc software, Ostend, Belgium). The χ^2^ Cochrane Q score was used to assess study heterogeneity. Owing to significant heterogeneity, a random‐effects model was used and a pooled comparison was not performed.

## Results

**Figure 1:**
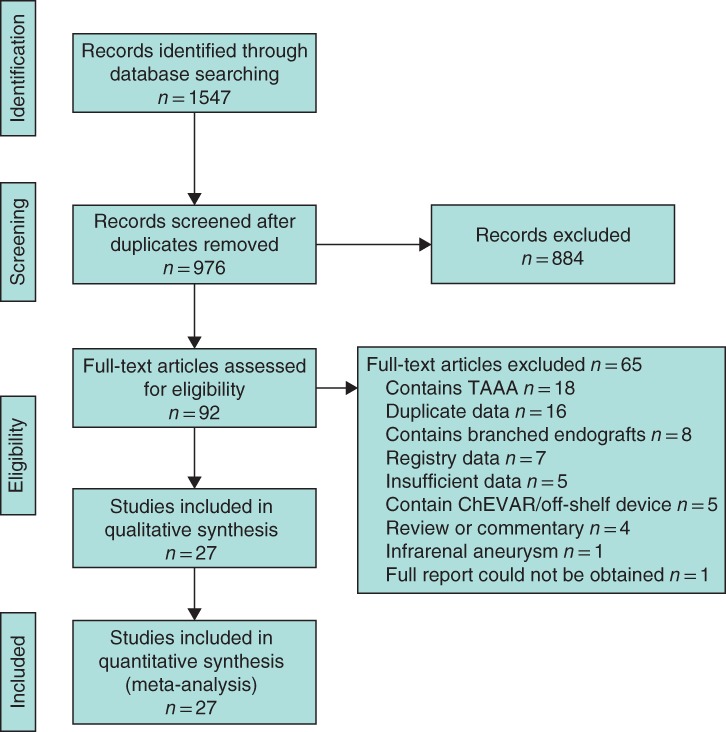
PRISMA flow diagram. TAAA, thoracoabdominal aortic aneurysm; ChEVAR, chimney endovascular aneurysm repair

The literature search identified 1547 potential articles. Following assessment this was refined to 27 studies, 14 involving FEVAR and 13 involving OSR, which were analysed using qualitative and quantitative methods. The PRISMA flow diagram is illustrated in *Fig*. 1. Funnel plots were used to assess for risk of publication bias (*Fig*. *S1*, supporting information).

### Study characteristics

Of the 14 studies involving FEVAR, the study designs included five case series^11^, ^12^, ^13^, ^14^, ^15^, four series within retrospective cohort studies comparing FEVAR with other endovascular interventions^7^, ^16^, ^17^, ^18^, three retrospective cohort studies that compared varying complexity of FEVAR^19^, ^20^, ^21^, one case–control study^22^ and a single non‐randomized trial^23^ (*Table* 1). Eleven single‐centre and three multicentre studies were included, with study sizes ranging from 20 to 384 patients, and follow‐up from 1 to 67 months. Three series identified were from the UK, the largest of these being that of Roy and colleagues^11^, involving 173 patients over a follow‐up period of 34 months.

**Table 1:** Design and baseline characteristics of fenestrated endovascular aneurysm repair studies

Reference	Study design	Country	Sites	*n*	Follow‐up (months)	Definition of early mortality	Definition of renal insufficiency	Aneurysm morphology
Oikonomou *et al.* ^19^	Retrospective cohort	Germany	1	141	33	30 days	Reduction eGFR > 30%	Short‐necked, juxtarenal and suprarenal
Roy *et al.* ^11^	Case series	UK	1	173	34	In‐hospital	> 50% rise in creatinine	n.s.
Wooster *et al.* ^16^	Retrospective cohort	USA	1	39	6·7	In‐hospital	n.s.	Extension to < 4 mm of renal artery and not above highest renal
Blankensteijn *et al.* ^12^	Case series	Netherlands	13	60	16·4	30 days	n.s.	Neck length < 15 mm and not extending above renal arteries
Katsargyris *et al.* ^20^	Retrospective cohort	Germany	1	384	20	30 days	> 30% rise in creatinine	Short‐necked, juxtarenal and suprarenal
Caradu *et al.* ^7^	Retrospective cohort	France	1	90	19	30 days or in‐hospital	‘Acute kidney injury’ (definition n.s.)	n.s.
Saratzis *et al.* ^22^	Case–control	UK	1	58	20	n.s.	> 50% rise in creatinine	Short‐necked and juxtarenal (suprarenal excluded)
Vemuri *et al.* ^13^	Case series	USA	7	57	1·75	30 days	n.s.	n.s.
Kristmundsson *et al.* ^14^	Case series	Sweden	1	54	67	30 days	> 30% decrease in eGFR	Short‐necked and juxtarenal (suprarenal excluded)
Grimme *et al.* ^15^	Case series	Netherlands	1	138	13	30 days	> 30% decrease in eGFR	Short‐necked, juxtarenal and suprarenal
Banno *et al.* ^17^	Retrospective cohort	France	1	80	14	30 days	> 50% rise in creatinine	Short‐necked and juxtarenal (suprarenal excluded)
Oderich *et al.* ^23^	Prospective non‐randomized	USA	14	67	37	30 days	> 30% decrease in eGFR in two tests	Neck length ≥ 4 mm and ≤ 15 mm
Perot *et al.* ^18^	Retrospective cohort	France	1	115	20	30 days	Creatinine clearance < 60 ml/min	Suprarenal and juxtarenal
Manning *et al.* ^21^	Retrospective cohort	UK	1	20	1	n.s.	n.s.	n.s.

eGFR, Estimated glomerular filtration rate; n.s., not stated.

Of the 13 studies involving OSR, the study designs included six case series^24^, ^25^, ^26^, ^27^, ^28^, ^29^, four retrospective cohort studies comparing OSR with FEVAR^30^, ^31^, ^32^, ^33^, and three retrospective cohort studies comparing OSR of juxtarenal and infrarenal aneurysms^34^, ^35^, ^36^ (*Table* 2). Twelve single‐centre studies and one multicentre study were included, with study sizes ranging from 31 to 214 patients and follow‐up from 1 to 66 months.

**Table 2:** Design and baseline characteristics of open surgical repair studies

Reference	Study design	Country	Sites	*n*	Follow‐up (months)	Definition of early mortality	Definition of renal insufficiency	Definition of juxtarenal/suprarenal aneurysm
Van Lammeren *et al.* ^24^	Case series	Netherlands	1	214	21·6	30 days	n.s. (new‐onset eGFR < 15 ml per min per l.73 m^2^ or dialysis recorded as renal failure)	Cross‐clamp above ≥ 1 renal artery
Shahverdyan *et al.* ^30^	Retrospective cohort	Germany	1	34	66	30 days	> 50% rise in creatinine and/or > 25% decrease in eGFR	Suprarenal or higher cross‐clamp
Barillà *et al.* ^31^	Retrospective cohort	Italy	1	50	n.s.	30 days	> 30% rise in creatinine	*
Canavati *et al.* ^32^	Retrospective cohort	UK	1	54	1	30 days or in‐hospital	> 50% rise in creatinine	Aneurysm neck < 10 mm
Dubois *et al.* ^25^	Case series	Canada	1	169	1	In‐hospital	> 25% decrease in eGFR	Suprarenal or higher cross‐clamp
Tsai *et al.* ^26^	Case series	USA	1	199	56	30 days	Creatinine rise > 0·5 mg/dl over baseline and > 1·5 mg/dl	Suprarenal or higher cross‐clamp
Donas *et al.* ^33^	Retrospective cohort	Germany	1	31	14·1	30 days	Doubling of baseline creatinine or decrease in eGFR > 50%	Suprarenal or higher cross‐clamp
Jeyabalan *et al.* ^27^	Case series	USA and South Korea	2	184	26·4	n.s.	Creatinine rise > 0·5 mg/dl	Cross‐clamp above ≥ 1 renal artery
Landry *et al.* ^34^	Retrospective cohort	USA	1	82	1	30 days	Creatinine rise > 0·5 mg/dl	Suprarenal or higher cross‐clamp
Chong *et al.* 35	Retrospective cohort	USA	1	171	56·7	30 days	eGFR < 30 ml per min per 1·73 m^2^ and > 20% decrease in eGFR	Suprarenal or higher cross‐clamp
Knott *et al.* ^28^	Case series	USA	1	126	48	30 days	Creatinine rise > 0·5 mg/dl	Cross‐clamp above ≥ 1 renal artery
Pearce *et al.* ^29^	Case series	USA	1	150	32	30 days or in‐hospital	> 20% rise in creatinine and level > 1·5 mg/dl in men, > 1·3 mg/dl in women	Cross‐clamp above ≥ 1 renal artery
Ockert *et al.* ^36^	Retrospective cohort	Germany	1	35	27·6	In‐hospital	Doubling of baseline creatinine or creatinine level > 1·3 mg/dl	Suprarenal or higher cross‐clamp

* Hostile neck defined as: length < 10 mm, angle ≥ 60°, ≥ 50% calcified neck, diameter > 31 mm, > 50% circumferential thrombus, reverse taper configuration. eGFR, Estimated glomerular filtration rate; n.s., not stated.

Qualitative assessment revealed a large proportion of single‐centre case series with a lack of internal controls. The included retrospective cohort studies have a high risk of selection and recorder bias. The significant variability in length of follow‐up affects the validity of a number of secondary outcomes, in particular the incidence of reintervention. Variability of outcome reporting, particularly with respect to the definitions of renal insufficiency, exists within the selected series. Overall the quality of available evidence was low.

### Baseline characteristics

A total of 2975 patients were included, 1476 who underwent FEVAR and 1499 who had OSR. Baseline characteristics of the two groups are shown in *Table* 3. Patients undergoing FEVAR had more medical co‐morbidities. Pre‐existing renal dysfunction was twice as high in the FEVAR cohort; these patients also displayed higher rates of ischaemic heart disease and pulmonary dysfunction. The breakdown of study design and patient characteristics is shown in *Tables* 1 and 2.

**Table 3:** Summary of baseline characteristics

	FEVAR	OSR
Age (years)*	73·2(1·4)	72·1(2·5)
Aneurysm diameter (cm)*	6·1(0·3)	6·3(0·4)
Renal dysfunction	37·8	16·7
Ischaemic heart disease	54·5	49·5
Pulmonary dysfunction	39·4	31·9
Diabetes	17·7	13·7

Values are percentages unless indicated otherwise;

* values are mean(s.d.). FEVAR, fenestrated endovascular aneurysm repair; OSR, open surgical repair.

### Mortality

**Figure 2:**
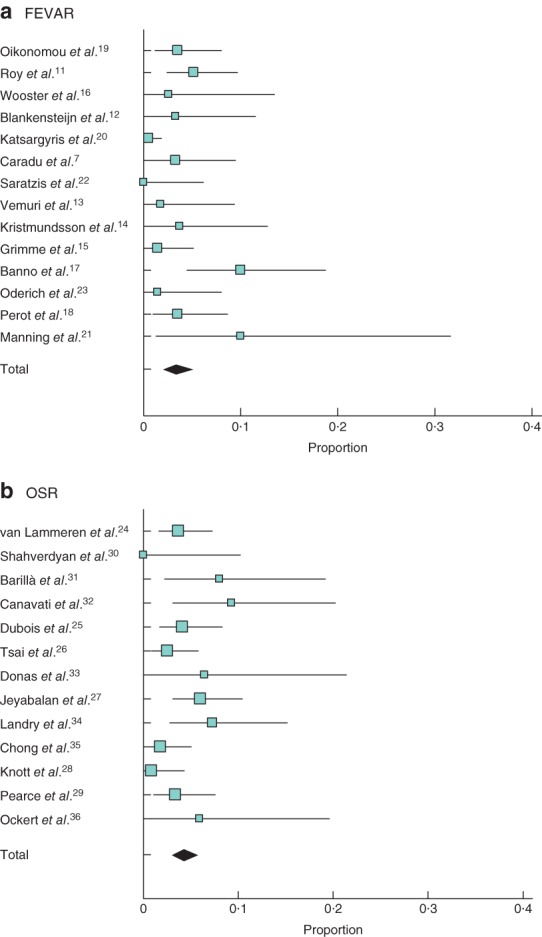
Thirty‐day/in‐hospital mortality after **a** fenestrated endovascular aneurysm repair (FEVAR) and **b** open surgical repair (OSR). A random‐effects model was used for meta‐analysis. Pooled rate of early postoperative mortality: **a** 3·3 (95 per cent c.i. 2·0 to 5·0) per cent; **b** 4·2 (2·9 to 5·7) per cent

The pooled rate of early postoperative mortality following FEVAR was 3·3 (95 per cent c.i. 2·0 to 5·0) per cent, compared with 4·2 (2·9 to 5·7) per cent after OSR (*Fig*. 2). Estimated long‐term survival was similar for FEVAR and OSR (*Tables* 4 and 5).

**Table 4:** Estimated long‐term survival after fenestrated endovascular aneurysm repair

	Survival after FEVAR (%)				
	1 year	2 years	3 years	4 years	5 years
Oikonomou *et al.* 19	85·1	–	75·8	–	–
Blankensteijn *et al.* ^12^	91·4	89·5	86·3	–	–
Katsargyris *et al.* ^20^ (standard FEVAR)	95	–	83·4	–	–
Katsargyris *et al.* ^20^ (complex FEVAR)	94	–	89·4	–	–
Caradu *et al.* ^7^	91·4	82·1	–	–	–
Kristmundsson *et al.* ^14^	93	–	76	–	60
Grimme *et al.* ^15^	89·2	83·2	71·9	62·5	53·3
Banno *et al.* ^17^	83·9	77·3	–	–	–
Oderich *et al.* ^23^	97	95·2	90·7	90·7	90·7
Perot *et al.* ^18^	93·9	–	–	–	–

FEVAR, fenestrated endovascular aneurysm repair.

**Table 5:** Estimated long‐term survival following open surgical repair

	Survival after OSR (%)				
	1 year	2 years	3 years	4 years	5 years
Shahverdyan *et al.* ^30^	90·3	90·3	–	–	–
Barillà *et al.* ^31^	90	84	74	70	65
Tsai *et al.* ^26^	91·5	87·1	82·5	79·2	74·2
Chong *et al.* ^35^	–	–	–	–	67·7
Knott *et al.* ^28^	94	–	78	–	64
Pearce *et al.* ^29^	88	82	78	72	69

OSR, open surgical repair.

### Postoperative renal insufficiency

**Figure 3:**
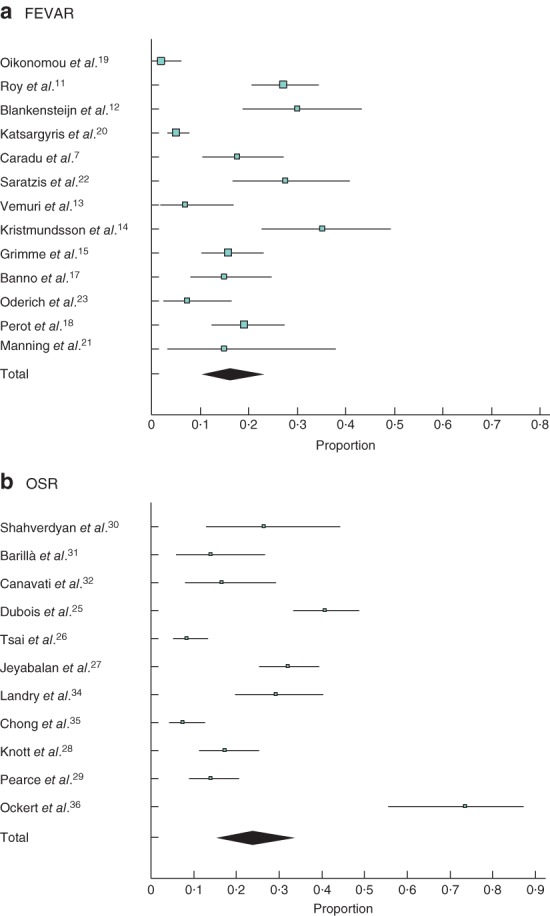
Renal insufficiency after **a** fenestrated endovascular aneurysm repair (FEVAR) and **b** open surgical repair (OSR). A random‐effects model was used for meta‐analysis. Pooled rate of postoperative renal insufficiency: **a** 16·2 (95 per cent c.i. 10·4 to 23·0) per cent; **b** 23·8 (15·2 to 33·6) per cent

After FEVAR, the pooled rate of postoperative renal insufficiency was 16·2 (95 per cent c.i. 10·4 to 23·0) per cent, compared with 23·8 (15·2 to 33·6) per cent after OSR (*Fig*. 3). The pooled rate of permanent dialysis was 0·8 (0·4 to 1·4) and 1·7 (1·0 to 2·5) per cent respectively.

### Major postoperative complications

Following FEVAR, the pooled major complication rate was 23·1 (95 per cent c.i. 16·8 to 30·1) per cent, compared with 43·5 (34·4 to 52·8) per cent after OSR. The rate of cardiac complications was 3·9 (1·8 to 6·9) and 13·4 (9·6 to 17·6) per cent respectively. Cardiac complications included myocardial infarction, cardiac arrhythmia and cardiac failure; however, this varied by study. Rates of myocardial infarction following FEVAR were 1·9 (0·4 to 4·7) per cent *versus* 5·8 (3·6 to 8·4) per cent after OSR. The pooled rate of mesenteric ischaemia was 2·2 (1·1 to 3·6) and 2·3 (1·3 to 3·6) per cent respectively.

### Reintervention

**Figure 4:**
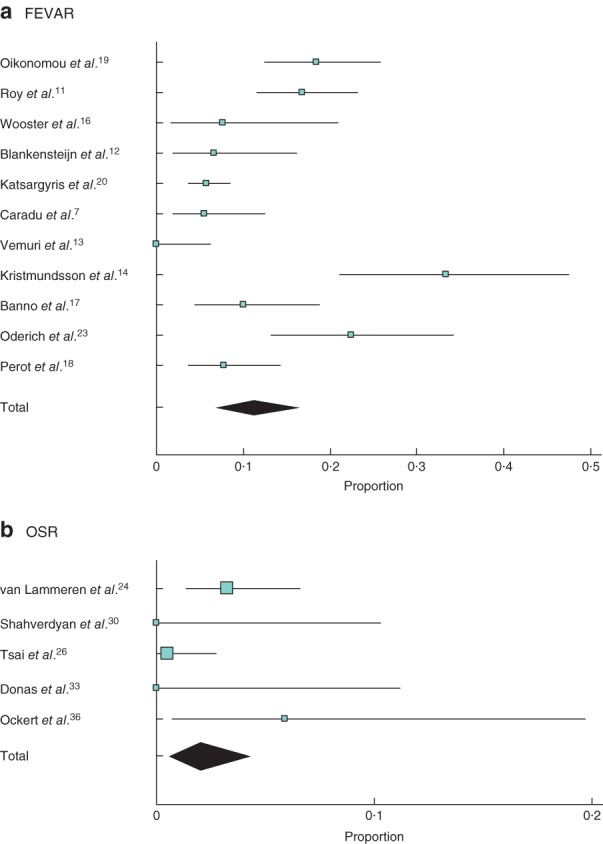
Late (more than 30 days) reintervention after **a** fenestrated endovascular aneurysm repair (FEVAR) and **b** open surgical repair (OSR). A random‐effects model was used for meta‐analysis. Late reintervention rate: 11·1 (6·7 to 16·4) per cent; **b** 2·0 (0·6 to 4·3) per cent

The overall rate of early reintervention (at less than 30 days) after FEVAR was 6·1 (95 per cent c.i. 3·2 to 9·8) per cent, compared with 7·4 (4·7 to 10·7) per cent after OSR. The rate of late reintervention following FEVAR was higher than that after OSR: 11·1 (6·7 to 16·4) and 2·0 (0·6 to 4·3) per cent respectively (*Fig*. 4), at a mean follow‐up of 21·6 and 31·1 months respectively.

### Rates of endoleak and occlusion after FEVAR

**Figure 5:**
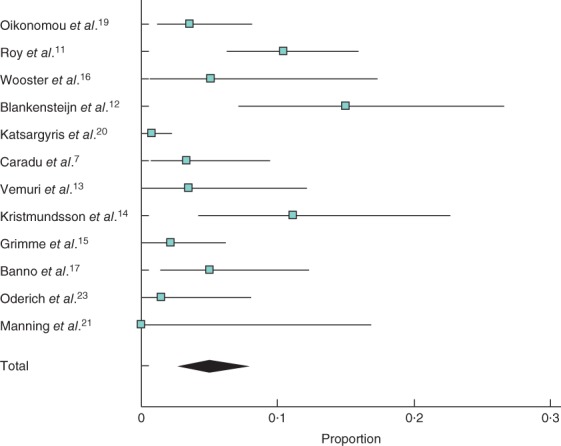
Combined rates of type I and type III endoleak. A random‐effects model was used for meta‐analysis. Incidence of type I/III endoleak: 4·9 (95 per cent c.i. 2·6 to 7·9) per cent

Overall, the incidence of type I/III endoleak was 4·9 (95 per cent c.i. 2·6 to 7·9) per cent (*Fig*. 5). Rates of target vessel preservation and long‐term patency are shown in *Table* 6. Target vessel preservation was high, with rates of occlusion during follow‐up between 2 and 4 per cent.

**Table 6:** Target vessel preservation (excluding vessels targeted by scallops)

	No. of patients	No. of vessels targeted (fenestrations/stents)	Mean no. of fenestrations per patient	No. of vessels stented successfully	Operative target vessel preservation rate (%)	Stenosis requiring reintervention	Target vessel occlusion during follow‐up	Target vessel occlusion rate during follow‐up (%)
Oikonomou *et al.* ^19^	141	403	2·86	n.s.	n.s.	12	8	n.s.
Roy *et al.* ^11^	173	446	2·58	440	98·7	n.s.	11	2·5
Wooster *et al.* ^16^	39	99	2·53	99	100	1	0	0
Blankensteijn *et al.* ^12^	60	140	2·33	136	97·1	0	4 (2 treated successfully)	2·9
Katsargyris *et al.* 20	384	n.s.	n.s.	n.s.	n.s.	n.s.	11	n.s.
Caradu *et al.* ^7^	90	198	2·2	196	99·0	n.s.	n.s.	n.s.
Saratzis *et al.* ^22^	58	150	2·59	n.s.	n.s.	n.s.	4 (of 116 followed)	3·5
Vemuri *et al.* ^13^	57	120	2·1	117	97·5	0	0	0
Kristmundsson *et al.* 14	54	96	1·78	n.s.	98	n.s.	n.s.	n.s.
Grimme *et al.* ^15^	138	254	1·84	249	98·0	n.s.	10	3·9
Banno *et al.* ^17^	80	194	2·42	189	97·4	n.s.	n.s.	n.s.
Oderich *et al.* ^23^	67	127 (stented target vessels)	1·90	127	100	11	4	3·1
Manning *et al.* ^21^	20	47 (scallops included)	2·35	65	95·6	n.a.	n.a.	n.a.

n.s., Not stated; n.a., not applicable.

### Subgroup analysis by complexity of FEVAR

Four^11^, ^19^, ^20^, ^21^ of the 13 studies provide sufficient data to compare the outcomes of FEVAR according to anatomical complexity. Patients were divided into two groups according to the level of vessels that were stented. Standard FEVAR was defined as stenting of the renal arteries with or without a scallop for the SMA, and complex FEVAR was defined as stenting of the renal arteries as well as the SMA and/or coeliac trunk. The groups consisted of 337 and 381 patients respectively. The data suggested a trend towards increased mortality with more complex repair, although this was not statistically significantly different (relative risk (RR) 1·38, 95 per cent c.i. 0·37 to 5·20; *P* = 0·634). There was no difference in technical success (RR 1·19, 0·95 to 1·01) or major complication (RR 1·19, 0·75 to 1·89) rates. Spinal cord ischaemia occurred only in patients who had undergone complex FEVAR (RR 4·71, 0·84 to 26·39), although this difference was not significantly different (*P* = 0·077). Rates of target vessel preservation and patency, and rates of endoleak were both reported inconsistently among studies. Reintervention rates were no different between standard and complex FEVAR groups (RR 0·90, 0·61 to 1·33; *P* = 0·343).

## Discussion

RCTs^10^, ^37^ have shown that the early safety profile of EVAR is better than that of OSR in the treatment of infrarenal AAA. A recent systematic review^38^ of these trials found the postoperative mortality rate associated with EVAR and OSR for infrarenal aneurysm to be 1·3 and 4·7 per cent respectively. This difference in early safety profile may not translate to the endovascular management of complex AAA. This review found similar mortality figures for FEVAR and OSR. These findings are supported by a previous review by Rao and colleagues^39^, which found a postoperative mortality rate of 4·1 per cent after both FEVAR and OSR. This may suggest that the physiological effects of treating the visceral segment are greater than those with a simple EVAR. It may also reflect the significantly co‐morbid patient cohort undergoing FEVAR in the studies analysed. Alternatively, it may reflect a lower than expected mortality rate in the OSR cohort, owing to the inclusion of trial data and publication bias, and may not reflect real‐world outcomes.

There is debate surrounding the long‐term renal implications of EVAR. FEVAR includes additional factors that place patients at risk of short‐ and long‐term renal complications: extensive instrumentation of the aorta and renal arteries to deploy the fenestrations, use of increased quantities of iodinated contrast, and increased risk of endoprosthesis failure necessitating further intervention. The renal risks of OSR stem from the ischaemic effects of suprarenal clamping, as well as the systemic response generated from significant surgery. The results of the present study suggest that, despite the large disparity in preoperative renal function, the incidence of renal impairment following FEVAR was lower than that after OSR. The incidence of permanent dialysis after OSR was 1·7 per cent, compared with 0·8 per cent after FEVAR. Long‐term renal outcomes were reported inconsistently in the included studies, and thus meaningful meta‐analysis cannot be performed. It should be noted that the heterogeneity of reporting and defining renal function limits the conclusions that can be drawn. Some studies reported renal insufficiency according to the RIFLE (risk, injury, failure, loss and end‐stage renal disease) criteria and defined it as either an increase in eGFR greater than 30 per cent from baseline or as a decrease in eGFR of more than 50 per cent from baseline, whereas others used use change in creatinine levels. It might be assumed that postoperative dialysis rates would provide a more reliable outcome, but studies varied when providing data regarding permanent or temporary dialysis; Pearce *et al.* ^29^ described ‘acute dialysis’, and did not state whether this was permanent or temporary.

High levels of technical success and target vessel preservation were seen throughout, with 11·1 per cent of patients requiring late reintervention after FEVAR. In the majority of cases, reintervention was required due to loss of target vessel patency, graft migration and endoleak. In contrast, only 2·0 per cent of patients required reintervention more than 30 days after OSR. There are, however, a number of factors to consider when interpreting this difference. Follow‐up of patients undergoing FEVAR is rigorous, resulting in comprehensive follow‐up data, yet those undergoing OSR rarely undergo long‐term surveillance. Long‐term complications following OSR are also poorly defined, resulting in under‐reporting of interventions such as incisional hernia repair and adhesiolysis for small bowel obstruction. Thus, these confounding factors should temper the conclusion that OSR has greater ‘reintervention free’ durability than FEVAR.

Long‐term survival was similar for OSR and FEVAR in this meta‐analysis. Comparison of long‐term outcomes between FEVAR and OSR is limited by selection bias, which renders the two cohorts mismatched in terms of co‐morbidities and surgical fitness. The difference in surgical suitability produces measurable differences in survival. The EVAR‐1 trial^9^ found estimated 4‐year survival to be 74 per cent, yet EVAR‐2^40^ (those unfit for open repair) found the estimated 4‐year survival rate to be significantly lower at 44 per cent. The true long‐term effects of each intervention may reflect a similar pattern seen when comparing standard EVAR and open repair: that the initial survival benefit of EVAR is eroded over time^41^. To compare long‐term survival between FEVAR and OSR an RCT would be required, in order to remove selection bias and allow comparison between two equally co‐morbid groups. Given the incidence of complex AAA and the different expertise of different centres, this will be challenging to instigate.

Despite increasing technical difficulty with complex FEVAR, no difference was noted in either technical success or mortality and morbidity.

Published registry data for FEVAR are available in the Global Collaborators on Advanced Stent‐Graft Techniques for Aneurysm Repair (GLOBALSTAR) database, the French registries WINDOWS (branched and fenestrated) and ANACONDA™ (Vascutek, Inchinnan, UK) Fenestrated Endografts in the Treatment of Complex Aortic Pathologies (EFEFA), and the American College of Surgeons' National Surgical Quality Improvement Program database. These provide data pertaining to the real‐world outcomes of complex EVAR^42^, ^43^, ^44^. Deery and colleagues^4^ published data from the Vascular Study Group of New England, comparing the outcomes of 1875 patients who had elective open repair of AAA between 2003 and 2011. Of these, 443 patients underwent repair of complex AAA, as defined by the requirement of a suprarenal or more proximal cross‐clamp of the aorta. The 30‐day mortality rate was 3·6 per cent. Although low, this was three times higher than the 1·2 per cent rate following repair of infrarenal AAA. Rates of postoperative morbidity reflected that found in the present review; renal impairment was 20 per cent, haemodialysis was required in 1·1 per cent and myocardial infarction occurred in 7 per cent of patients. In 2017, Ultee *et al.* ^45^ compared FEVAR with OSR for complex AAA within the National Surgical Quality Improvement Program (a database consisting of results from 200 hospitals across the USA). The 30‐day mortality rate was significantly higher after OSR: 6·6 per cent *versus* 3·4 per cent after FEVAR (*P* = 0·038). Although the incidence of AKI was lower in both groups compared with findings in the present review, the odds ratio for AKI following OSR was 4·8 (95 per cent c.i. 2·2 to 10·5; *P* < 0·001) compared with FEVAR^45^. In the UK, an analysis of Hospital Episode Statistics data gave a 30‐day mortality rate of 14·0 per cent after open suprarenal AAA repair^46^. The rate of freedom from all‐cause mortality was 78, 73, 67, 63 and 58 per cent in years 1, 2, 3, 4 and 5 after surgery respectively^46^. Inclusion was defined by procedural code ‘Replacement of aneurysmal segment of suprarenal abdominal aorta by anastomosis of aorta to aorta’; it therefore excluded juxtarenal AAA repair and may have included type IV TAAA, which may account for the significantly higher mortality described. Analysis of the GLOBALSTAR database was used to present the UK results of FEVAR between 2007 and 2010^42^. The perioperative mortality rate was 4·1 per cent, the early reintervention rate (within 30 days) 7 per cent, and the rate of intraoperative target vessel loss 0·6 per cent. The survival rate was 94, 91 and 89 per cent at 1, 2 and 3 years respectively^42^.

The difference in baseline characteristics due to the selection bias prevalent in clinical practice highlights the inherent problem with retrospective comparison of the results of FEVAR and OSR. The reporting of long‐term results, particularly with open series, is inconsistent as these patients are not placed under routine surveillance and do not routinely undergo postprocedural imaging; follow‐up imaging was performed in only one series^26^, rendering commentary on graft durability after open repair difficult. Given the low prevalence of late complications after OSR, this study does not support formal surveillance for this patient group, with intervention offered if and when symptoms develop. Inclusion and exclusion criteria with respect to aneurysm morphology, which varies between FEVAR studies, are an additional confounding factor, with some including only short‐necked infrarenal aneurysms and others including those that extend proximal to the coeliac trunk. The open series define the inclusion criteria differently according to the position of aortic cross‐clamp. Thus, the two cohorts are potentially distinct in their aneurysm characteristics.

Variability in reporting of outcomes, particularly renal outcomes, may have significantly affected the conclusions drawn. FEVAR is a complex endovascular procedure, associated with a significant learning curve. To reduce the effects of this, contemporary studies were used to remove study data obtained while the technique was being developed. It should be noted, however, that although current experience of OSR outweighs that of FEVAR, the tendency towards endovascular techniques results in a shortfall in trainee experience of OSR. Results of complex OSR have likely plateaued and may begin to see a downward trend^47^. In contrast, the learning curve continues with FEVAR, and with further innovation in stent design and fusion imaging, results will continue to improve. The majority of high‐volume, specialized centres are located outside the UK, raising the question of whether the results are generalizable to small centres in the UK.

Owing to the heterogeneity present, both between treatment groups as a result of sampling and selection bias and between study results within a single treatment arm (as demonstrated by χ^2^ Cochrane Q score), a pooled comparison was not performed. Ideally, a randomized trial would be conducted to determine definitively which procedure patients should be offered as a first‐line intervention. In the absence of such a study, all patients undergoing complex AAA repair should be entered into mandatory registries with an agreed set of co‐morbidity and outcome data to improve the analysis of real‐world outcomes in the future.

## Supporting information


**Fig. S1** Funnel plot of standard error by 30‐day mortality, FEVAR (above) and OSR (below)

## References

[1] Crawford ES , Beckett WC , Greer MS . Juxtarenal infrarenal abdominal aortic aneurysm. Special diagnostic and therapeutic considerations. Ann Surg 1986; 203: 661–670. 3521511 10.1097/00000658-198606000-00011 PMC1251200

[2] Fillinger MF , Greenberg RK , McKinsey JF , Chaikof EL ; Society for Vascular Surgery Ad Hoc Committee on TEVAR Reporting Standards. Reporting standards for thoracic endovascular aortic repair (TEVAR). J Vasc Surg 2010; 52: 1022–1033.e15. 20888533 10.1016/j.jvs.2010.07.008

[3] Bryce Y , Rogoff P , Romanelli D , Reichle R . Endovascular repair of abdominal aortic aneurysms: vascular anatomy, device selection, procedure, and procedure‐specific complications. Radiographics 2015; 35: 593–615. 25763741 10.1148/rg.352140045

[4] Deery SE , Lancaster RT , Baril DT , Indes JE , Bertges DJ , Conrad MF *et al.* Contemporary outcomes of open complex abdominal aortic aneurysm repair. J Vasc Surg 2016; 63: 1195–1200. 27109792 10.1016/j.jvs.2015.12.038

[5] England A , McWilliams R . Endovascular aortic aneurysm repair (EVAR). Ulster Med J 2013; 82: 3–10. 23620623 PMC3632841

[6] Hertault A , Haulon S , Lee JT . Debate: whether branched/fenestrated endovascular aneurysm repair procedures are better than snorkels, chimneys, or periscopes in the treatment of most thoracoabdominal and juxtarenal aneurysms. J Vasc Surg 2015; 62: 1357–1365. 26506275 10.1016/j.jvs.2015.07.001

[7] Caradu C , Morin J , Poirier M , Midy D , Ducasse E . Monocentric evaluation of chimney *versus* fenestrated endovascular aortic repair for juxtarenal abdominal aortic aneurysm. Ann Vasc Surg 2017; 40: 28–38. 28161566 10.1016/j.avsg.2016.09.013

[8] Li Y , Hu Z , Bai C , Liu J , Zhang T , Ge Y *et al.* Fenestrated and chimney technique for juxtarenal aortic aneurysm: a systematic review and pooled data analysis. Sci Rep 2016; 6: 20497. 26869488 10.1038/srep20497 PMC4751537

[9] EVAR trial participants . Endovascular aneurysm repair *versus* open repair in patients with abdominal aortic aneurysm (EVAR trial 1): randomised controlled trial. Lancet 2005; 365: 2179–2186. 15978925 10.1016/S0140-6736(05)66627-5

[10] Prinssen M , Verhoeven EL , Buth J , Cuypers PW , van Sambeek MR , Balm R *et al.*; Dutch Randomized Endovascular Aneurysm Management (DREAM)Trial Group. A randomized trial comparing conventional and endovascular repair of abdominal aortic aneurysms. N Engl J Med 2004; 351: 1607–1618. 15483279 10.1056/NEJMoa042002

[11] Roy IN , Millen AM , Jones SM , Vallabhaneni SR , Scurr JRH , McWilliams RG *et al.* Long‐term follow‐up of fenestrated endovascular repair for juxtarenal aortic aneurysm. Br J Surg 2017; 104: 1020–1027. 28401533 10.1002/bjs.10524 PMC5485015

[12] Blankensteijn LL , Dijkstra ML , Tielliu IF , Reijnen MM , Zeebregts CJ ; Dutch Fenestrated Anaconda Research Group. Midterm results of the fenestrated Anaconda endograft for short‐neck infrarenal and juxtarenal abdominal aortic aneurysm repair. J Vasc Surg 2017; 65: 303–310. 28029566 10.1016/j.jvs.2016.08.092

[13] Vemuri C , Oderich GS , Lee JT , Farber MA , Fajardo A , Woo EY *et al.* Postapproval outcomes of juxtarenal aortic aneurysms treated with the Zenith fenestrated endovascular graft. J Vasc Surg 2014; 60: 295–300. 24680241 10.1016/j.jvs.2014.01.071

[14] Kristmundsson T , Sonesson B , Dias N , Törnqvist P , Malina M , Resch T . Outcomes of fenestrated endovascular repair of juxtarenal aortic aneurysm. J Vasc Surg 2014; 59: 115–120. 24011738 10.1016/j.jvs.2013.07.009

[15] Grimme FA , Zeebregts CJ , Verhoeven EL , Bekkema F , Reijnen MM , Tielliu IF . Visceral stent patency in fenestrated stent grafting for abdominal aortic aneurysm repair. J Vasc Surg 2014; 59: 298–306. 24080136 10.1016/j.jvs.2013.08.005

[16] Wooster M , Tanious A , Patel S , Moudgill N , Back M , Shames M . Concomitant parallel endografting and fenestrated experience in a regional aortic center. Ann Vasc Surg 2017; 38: 54–58. 27793620 10.1016/j.avsg.2016.09.009

[17] Banno H , Cochennec F , Marzelle J , Becquemin JP . Comparison of fenestrated endovascular aneurysm repair and chimney graft techniques for pararenal aortic aneurysm. J Vasc Surg 2014; 60: 31–39. 24560863 10.1016/j.jvs.2014.01.036

[18] Perot C , Sobocinski J , Maurel B , Millet G , Guillou M , d'Elia P *et al.* Comparison of short‐ and mid‐term follow‐up between standard and fenestrated endografts. Ann Vasc Surg 2013; 27: 562–570. 23403332 10.1016/j.avsg.2011.11.047

[19] Oikonomou K , Kasprzak P , Schierling W , Kopp R , Pfister K . Graft complexity‐related outcomes of fenestrated endografting for abdominal aortic aneurysms. J Endovasc Ther 2017; 24: 230–236. 28205454 10.1177/1526602817691752

[20] Katsargyris A , Oikonomou K , Kouvelos G , Mufty H , Ritter W , Verhoeven ELG . Comparison of outcomes for double fenestrated endovascular aneurysm repair *versus* triple or quadruple fenestrated endovascular aneurysm repair in the treatment of complex abdominal aortic aneurysms. J Vasc Surg 2017: 66: 29–36. 28189357 10.1016/j.jvs.2016.11.043

[21] Manning BJ , Agu O , Richards T , Ivancev K , Harris PL . Early outcome following endovascular repair of pararenal aortic aneurysms: triple‐ *versus* double‐ or single‐fenestrated stent‐grafts. J Endovasc Ther 2011; 18: 98–105. 21314357 10.1583/10-3122.1

[22] Saratzis AN , Bath MF , Harrison SC , Sayers RD , Bown MJ . Impact of fenestrated endovascular abdominal aortic aneurysm repair on renal function. J Endovasc Ther 2015; 22: 889–896. 26359438 10.1177/1526602815605311

[23] Oderich GS , Greenberg RK , Farber M , Lyden S , Sanchez L , Fairman R *et al.*; Zenith Fenestrated Study Investigators. Results of the United States multicenter prospective study evaluating the Zenith fenestrated endovascular graft for treatment of juxtarenal abdominal aortic aneurysms. J Vasc Surg 2014; 60: 1420–1428.e5. 25195145 10.1016/j.jvs.2014.08.061

[24] van Lammeren GW , Ünlü Ç , Verschoor S , van Dongen EP , Wille J , van de Pavoordt ED *et al.* Results of open pararenal abdominal aortic aneurysm repair: single centre series and pooled analysis of literature. Vascular 2017; 25: 234–241. 27565511 10.1177/1708538116665268

[25] Dubois L , Durant C , Harrington DM , Forbes TL , Derose G , Harris JR . Technical factors are strongest predictors of postoperative renal dysfunction after open transperitoneal juxtarenal abdominal aortic aneurysm repair. J Vasc Surg 2013; 57: 648–654. 23312936 10.1016/j.jvs.2012.09.043

[26] Tsai S , Conrad MF , Patel VI , Kwolek CJ , LaMuraglia GM , Brewster DC *et al.* Durability of open repair of juxtarenal abdominal aortic aneurysms. J Vasc Surg 2012; 56: 2–7. 22534029 10.1016/j.jvs.2011.12.085

[27] Jeyabalan G , Park T , Rhee RY , Makaroun MS , Cho JS . Comparison of modern open infrarenal and pararenal abdominal aortic aneurysm repair on early outcomes and renal dysfunction at one year. J Vasc Surg 2011; 54: 654–659. 21620619 10.1016/j.jvs.2011.03.007

[28] Knott AW , Kalra M , Duncan AA , Reed NR , Bower TC , Hoskin TL *et al.* Open repair of juxtarenal aortic aneurysms (JAA) remains a safe option in the era of fenestrated endografts. J Vasc Surg 2008; 47: 695–701. 18272317 10.1016/j.jvs.2007.12.007

[29] Pearce JD , Edwards MS , Stafford JM , Deonanan JK , Davis RP , Corriere MA *et al.* Open repair of aortic aneurysms involving the renal vessels. Ann Vasc Surg 2007; 21: 676–686. 17923384 10.1016/j.avsg.2007.07.011

[30] Shahverdyan R , Majd MP , Thul R , Braun N , Gawenda M , Brunkwall J. F‐EVAR does not impair renal function more than open surgery for juxtarenal aortic aneurysms: single centre results. Eur J Vasc Endovasc Surg 2015; 50: 432–441. 26100450 10.1016/j.ejvs.2015.04.028

[31] Barillà D , Sobocinski J , Stilo F , Maurel B , Spinelli F , Haulon S. Juxtarenal aortic aneurysm with hostile neck anatomy: midterm results of minilaparotomy *versus* f‐EVAR. Int Angiol 2014; 33: 466–473. 25294289

[32] Canavati R , Millen A , Brennan J , Fisher RK , McWilliams RG , Naik JB *et al.* Comparison of fenestrated endovascular and open repair of abdominal aortic aneurysms not suitable for standard endovascular repair. J Vasc Surg 2013; 57: 362–367. 23044256 10.1016/j.jvs.2012.08.040

[33] Donas KP , Eisenack M , Panuccio G , Austermann M , Osada N , Torsello G . The role of open and endovascular treatment with fenestrated and chimney endografts for patients with juxtarenal aortic aneurysms. J Vasc Surg 2012; 56: 285–290. 22554426 10.1016/j.jvs.2012.01.043

[34] Landry G , Lau I , Liem T , Mitchell E , Moneta G . Open abdominal aortic aneurysm repair in the endovascular era: effect of clamp site on outcomes. Arch Surg 2009; 144: 811–816. 19797104 10.1001/archsurg.2009.157

[35] Chong T , Nguyen L , Owens CD , Conte MS , Belkin M . Suprarenal aortic cross‐clamp position: a reappraisal of its effects on outcomes for open abdominal aortic aneurysm repair. J Vasc Surg 2009; 49: 873–880. 19233593 10.1016/j.jvs.2008.10.057

[36] Ockert S , Schumacher H , Böckler D , Malcherek K , Hansmann J , Allenberg J . Comparative early and midterm results of open juxtarenal and infrarenal aneurysm repair. Langenbecks Arch Surg 2007; 392: 725–730. 17242895 10.1007/s00423-006-0141-6

[37] Greenhalgh RM , Brown LC , Kwong GP , Powell JT , Thompson SG ; EVAR trial participants. Comparison of endovascular aneurysm repair with open repair in patients with abdominal aortic aneurysm (EVAR trial 1), 30‐day operative mortality results: randomised controlled trial. Lancet 2004; 364: 843–848. 15351191 10.1016/S0140-6736(04)16979-1

[38] Stather PW , Sidloff D , Dattani N , Choke E , Bown MJ , Sayers RD . Systematic review and meta‐analysis of the early and late outcomes of open and endovascular repair of abdominal aortic aneurysm. Br J Surg 2013; 100: 863–872. 23939855 10.1002/bjs.9247

[39] Rao R , Lane TR , Franklin IJ , Davies AH . Open repair *versus* fenestrated endovascular aneurysm repair of juxtarenal aneurysms. J Vasc Surg 2015; 61: 242–255. 25240242 10.1016/j.jvs.2014.08.068

[40] EVAR trial participants . Endovascular aneurysm repair and outcome in patients unfit for open repair of abdominal aortic aneurysm (EVAR trial 2): randomised controlled trial. Lancet 2005; 365: 2187–2192. 15978926 10.1016/S0140-6736(05)66628-7

[41] Patel R , Sweeting MJ , Powell JT , Greenhalgh RM ; EVAR trial investigators . Endovascular *versus* open repair of abdominal aortic aneurysm in 15‐years' follow‐up of the UK endovascular aneurysm repair trial 1 (EVAR trial 1): a randomised controlled trial. Lancet 2016; 388: 2366–2374. 27743617 10.1016/S0140-6736(16)31135-7

[42] British Society for Endovascular Therapy and the Global Collaborators on Advanced Stent‐Graft Techniques for Aneurysm Repair (GLOBALSTAR) Registry. Early results of fenestrated endovascular repair of juxtarenal aortic aneurysms in the United Kingdom. Circulation 2012; 125: 2707–2715. 22665884 10.1161/CIRCULATIONAHA.111.070334

[43] Michel M , Becquemin JP , Clément MC , Marzelle J , Quelen C , Durand‐Zaleski I ; WINDOW Trial Participants . Editor's choice ‐ Thirty day outcomes and costs of fenestrated and branched stent grafts *versus* open repair for complex aortic aneurysms. Eur J Vasc Endovasc Surg 2015; 50: 189–196. 26100447 10.1016/j.ejvs.2015.04.012

[44] Tsilimparis N , Perez S , Dayama A , Ricotta JJ 2nd. Endovascular repair with fenestrated‐branched stent grafts improves 30‐day outcomes for complex aortic aneurysms compared with open repair. Ann Vasc Surg 2013; 27: 267–273. 23403330 10.1016/j.avsg.2012.05.022

[45] Ultee KHJ , Zettervall SL , Soden PA , Darling J , Verhagen HJM , Schermerhorn ML . Perioperative outcome of endovascular repair for complex abdominal aortic aneurysms. J Vasc Surg 2017; 65: 1567–1575. 28216344 10.1016/j.jvs.2016.10.123 PMC5438879

[46] Karthikesalingam A , Holt PJ , Patterson BO , Vidal‐Diez A , Sollazzo G , Poloniecki JD *et al.* Elective open suprarenal aneurysm repair in England from 2000 to 2010 an observational study of hospital episode statistics. PLoS One 2013; 8: e64163. 23717559 10.1371/journal.pone.0064163 PMC3662715

[47] Dua A , Koprowski S , Upchurch G , Lee CJ , Desai SS . Progressive shortfall in open aneurysm experience for vascular surgery trainees with the impact of fenestrated and branched endovascular technology. J Vasc Surg 2017; 65: 257–261. 27743805 10.1016/j.jvs.2016.08.075

