# Supplementary material for: Meta‐analysis of fenestrated endovascular aneurysm repair versus open surgical repair of juxtarenal abdominal aortic aneurysms over the last 10 years
Source: BJS Open. 2019 May 17;3(5):572–84. doi: 10.1002/bjs5.50178 (PMC6773647; doi:10.1002/bjs5.50178)
Supplement: Supplementary file 1 — Fig. S1 Funnel plot of standard error by 30‐day mortality, FEVAR (above) and OSR (below) [file BJS5-3-572-s001.docx]

**BJS5_50178**

**Meta-analysis of fenestrated endovascular aneurysm repair *versus* open surgical repair of juxtarenal abdominal aortic aneurysms over the last 10 years**

**A. D. Jones, M. A. Waduud, P. Walker, D. Stocken, M. A. Bailey and D. J. A. Scott**

**Fig. S1** Funnel plot of standard error by 30-day mortality, FEVAR (above) and OSR (below)
